# Relationship of morphometrics, total carotenoids, and total lipids with activity and sexual and spatial features in *Euphausia superba*

**DOI:** 10.1038/s41598-020-69780-8

**Published:** 2020-08-06

**Authors:** Jaime Färber Lorda, Hubert J. Ceccaldi

**Affiliations:** 1 Departamento de Ecología, Centro de Investigación Científica y de Educación Superior de Ensenada, Ensenada, Baja California México; 2 Académie des Sciences, Lettres et Arts de Marseille, Marseille, France

**Keywords:** Developmental biology, Ecology

## Abstract

Morphological differences associated with sex or stage, together with total lipids and carotenoids, were studied in *Euphausia superba* as possible indicators of physiological condition. *E. superba* displays sexual dimorphism during growth. A group of mature males, called Males II herein, has a greater abdominal length, suggesting that they are faster swimmers, a feature implying higher metabolic rates and a higher demand for protecting pigments like carotenoids. Mature Males II have proportionally lower lipids but higher total lipid-soluble carotenoids, a counterintuitive finding. Males II also have bigger eyes. Significant regressions with carotenoids were found for wet weight, abdominal length, and eye diameter. On a spatial analysis, population composition reflects reproductive activity. Males II would be in search of females for fecundation and, thus, are dominant in some areas. The PCA analysis of 10 allometric and biochemical variables show a distinct Males II group differing in morphology, carotenoids, and lipid contents. The carotenoid:lipid ratio was highest for Males II, supporting the hypothesis of the role of carotenoids in the activity of the species. Mature males may experience physiological stress during reproduction and probably die shortly afterwards. A relationship between activity, morphometrics, and carotenoid content seems evident, deserving further investigation.

## Introduction

The Antarctic krill, *Euphausia superba,* has been the focus of multiple studies in the past because of its central role in the trophic web in the Antarctic Ocean. It has been found that during its ontogenetic development, *E. superba* undergoes a morphological transformation related primarily to sexual maturity^1–9^.

For *E. superba,* Miller (1983) found a significant difference between sexes, as well as between mature and immature females, in the slope of the regression between total length and carapace length^5^. Färber Lorda (1986, 1990) found an ontogenetic morphometric differentiation in *E. superba* and *Thysanoessa macrura*, and proposed a Differentiation Index (DI) to classify groups of individuals according to sex or age based on morphological traits^7,8^. Two groups of males were identified, hereafter referred to as Males I and Males II, which are related to the ratios between total body length, carapace length, and abdominal length^8^. In order to determine the longevity of *E. superba* and *T. macrura—*the dominant and coexisting species during summer in the southern part of the Indian Ocean, a multivariate study including morphological information showed that total lipids and total carotenoids also differ in quantity across development stages, assuming a 4-year life cycle based on morphometric, lipid, and carotenoid data, and a two-year life cycle for *T. macrura*. Males II have a lower lipid content versus all other sex groups or development stages^9^. Three age groups were separated when lipofuscines (age pigments) and morphometric variables were analysed, also showing that these groups could be separated based on morphometric measurements alone, indicating a morphological differentiation in Antarctic krill^10^.

The allometric relationship of total length vs. wet weight for Males II showed a steeper slope and a higher condition coefficient relative to the other sex or development stages; however, a spatial analysis revealed geographic differences beyond those related to sex or development stage^11^. In another paper, Färber Lorda et al. also showed that, spatially, the composition of populations was a key driver of mean total lipid content and mean DI^12^. An important implication of these differences is that animals in continuous movement, like krill^13^, will have faster swimming capacities. Males II have a longer abdomen, suggesting that they are better or faster swimmers^8^. In *E. superba,* a stronger but slower beating of pleopods was found in males, as compared to females^14^. Thus, we assumed a faster swimming capacity in mature Males II, which are expected to be more active in search of females for fecundation during the reproductive season in summer. The population sex ratio was found to be largely influenced by the swimming capacities of males and females, and may contribute to the segregation of different sexes or development stages within swarms of *E. superba*^13,15,16^; a faster growth rate was observed in males than in females during the Antarctic summer^17^. A reanalysis of body shrinkage during winter in *E. superba* in its natural habitat as compared to animals reared in aquaria^18^ found that it occurred only in females, but not in males^19^.

Lipids in krill have been widely studied, finding important differences between sexes^11,12,20–27^, especially during the reproductive season. However, Cripps et al.^28^, analysing fatty acids in both phytoplankton and *E. superba*, concluded that diet is likely more important than sex or development stage to determine fatty acid composition^28^. Geographic distribution is also related to differences in the biochemical composition of krill, especially as regards lipids and population structure^13,25^. Bouyancy has been studied in zooplankton, and it is hypothesised that lipids contribute to the buoyancy capacity of many zooplankton species, particularly *E. superba.* Water temperature plays a central role, as the proportion of lipids in solid phase contributes to buoyancy. The ratio of solid and liquid lipids constitutes a key element for the maintenance of krill at a depth that provides sufficient food and other favourable environmental conditions^29^.

Krill performs vertical migrations like most zooplankton, which apparently is a defence mechanism to avoid predation and to remain within the depth range where food is available^30^. This behaviour also confers protection from damaging light irradiance^31,32^, in addition to the synthesis of carotenoids–mostly astaxanthin, a potent antioxidant^33–41^.

Carotenoids in euphausiids were first studied by Wagner (1939, in Mauchline and Fisher, 1969) using euphausiids from baleen-whale stomach contents^42^. This author claimed that ß-carotene was converted into vitamin A in the stomach of baleen whales; further work by Kon and Thompson showed that the carotenoid astaxanthin was the dominant pigment in euphausiids, together with vitamin A^33,34^. Fisher et al. studied the evolution of the pigments vitamin A and astaxanthin in *Meganyctiphanes norvegica* and *Thysanoessa raschii* in relation to body weight, reporting higher concentrations in smaller animals^43^. These authors also reported higher levels in larval stages of both species and higher concentrations of these pigments in the eyes, a finding also supported by Czerpak et al.^38^. In a seasonal study, Fisher et al. found that both vitamin A and astaxanthin increased with body weight; however, fluctuations were also important, being related to higher phytoplankton availability, especially diatoms, with higher values in spring^44^. The exposure of krill to different light radiation intensities during vertical migrations and seasonal changes in light intensity under pack-ice produce important seasonal changes in the concentration of carotenoids and the colour of individuals, as shown by Auerswald et al. for *E. superba*^41^. However, former studies showed that krill is highly sensitive to ultraviolet radiation, which may lead to DNA damage^45^ and higher mortality, as shown by Newman et al.^46^, thus producing an avoidance reaction in *E. superba*^47^.

Shifts in colour have been classified as physiological and morphological changes^41^. In krill, physiological colour change takes place readily and occurs in specialized cells, chromatophores, which can adopt different colours. Morphological changes could be due to either an increase or a decrease in pigment concentration, the amount of chromatophores present, or a combination of both^41^. The Babak Law states that the number of chromatophores or the concentration of pigments either increase or decrease in relation to prolonged light periods lasting from days to months^48^. Crustaceans do not synthesize carotenoids, so they have to feed on them; once consumed, carotenoids are mostly transformed into astaxanthin^37^.

This study determined the total carotenoid and total lipid contents, along with morphometric measurements, in *E. superba* individuals of different sex and development stages, from various locations across the Antarctic Ocean. These individuals were collected over a short time during summer, mostly after the reproductive season, a key period for the metabolism of lipids and carotenoids according to previous results by other authors^18,26,35^.

## Material and methods

**Figure 1:**
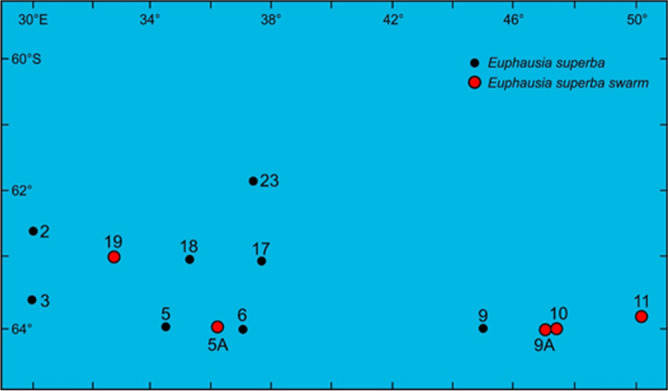
Stations sampled during the cruise FIBEX MD 25 in February 1981.

Samples of Antarctic krill were collected in the southern part of the Indian Ocean during the FIBEX MD 25 cruise on board R. V. Marion Dufresne in the summer (February, 1981), using an RMT net with 5 mm mesh size and 2 mm in the cod end. Samples were collected from each of 15 stations at noon and midnight, following echo-sounding signals; some samples were collected following swarm detections at given stations (Fig. 1). The specimens sampled were individually frozen at − 70 °C on board and transported to the laboratory. Once thawed, individuals were measured to the nearest 0.01 mm under a dissecting microscope. Samples were well-preserved, with no apparent damage and with turgescent eyes. This preservation method also maintained the specimens in good condition to perform the lipid and carotenoid analyses on the same individuals. The measurements performed were eye diameter, carapace length, total length, and wet weight, as described in Färber Lorda^7,8,11^; abdominal length was determined as the difference between total and carapace length. In the laboratory, each individual was weighed and measured to calculate the Differentiation Index (DI; Färber Lorda, 1986, 1990)^7,8^ as follows:

$${\text{DI}} = {\text{Total}}\;{\text{Length}}/\left( {{\text{Abdominal}}\;{\text{Length}} - {\text{Cephalothorax}}\;{\text{Length}}} \right)$$

DI values for the different sex or stage groups are shown in Table 1.

**Table 1:** Differentiation index (DI) of *Euphausia superba* by sex or stage; n shown in brackets in bold.

Sex or stage	Differentiation index
Juveniles **(44)**	4.506 ± 0.039
Mature Females **(19)**	5.839 ± 0.197
Spent Females **(14)**	5.703 ± 0.211
Males I **(29)**	4.334 ± 0.073
Males II **(13)**	3.180 ± 0.047

Afterwards, lipid extraction was performed and total carotenoids were estimated.

### Carotenoids and lipids

A pool of animals was first homogenized on a small volume of distilled water, after which the homogenate was split into two portions; lipids were extracted with the Bligh and Dyer^49^ method in one portion and with acetone in the other. The absorbance peak of both samples was compared on a scan from 300 to 700 nm, showing almost identical profiles and a similar peak with both extraction methods (Blight and Dyer, at 485 nm; acetone, at 490 nm); the experience was repeated three times with the same results. Total carotenoids were evaluated using the lipid extract read in a spectrophotometer at 485 nm; carotenoids concentration was calculated with the formula by Jackowska et al.^50^ as follows:

$${\text{C}}_{{\text{a}}} = \left( {\left( {\text{E}} \right)\left( {\text{v}} \right){ 1}0,000} \right)/\left( {\left( {{\text{E1}}\% } \right)\left( {\text{D}} \right)\left( {\text{W}} \right)} \right)$$

where C_a_ = carotenoids, in µg/gr (wet weight), E = absorbance of the sample, v = Extraction volume, D = width of the spectrophometer cuvette (1 cm), W = wet weight of the sample

$${\text{E1}}\% \, = {\text{ Extinction}}\;{\text{coefficient}}\;{\text{of}}\;{\text{the}}\;{\text{solution}}$$

Total carotenoids were calculated in µg per individual and in grams per individual. Lipids were first extracted from animals using the method of Bligh and Dyer^49^, followed by the colorimetric method of Pande et al.^51^ using tripalmitin as standard, as mentioned in Färber Lorda^7,9^; total lipids were expressed as mg per animal and as % wet weight. Data were processed and figures were plotted using the Sigmaplot program. These data were partially published elsewhere aiming to understand the longevity of *E. superba*^9^. Carotenoid data per individual or per g were tested for normality; they all showed a normal distribution for each sex or development stage, but for the entire sample, only the data expressed as carotenoids per g showed a normal distribution. ANOVA and regression analyses were performed on the data; for ANOVA, α was set at 95% (Table 3). When data were not normally distributed (*P* < 0.005), a Kruskall Wallis test was performed (*P* < 0.001) and Mann–Whitney’s U was calculated for each paired group of sex or development stage. When the paired data were normally distributed, the Student’s *t*-test was run; these tests were performed on the three main variables analysed (Results shown in Table 3). Lipid data expressed as % were arc-sin transformed and a Krukall-Wallis test was performed (*P* < 0.001). Power regression analyses were performed occasionally on non-normally distributed data; they are shown with their 95% confidence intervals. Cohen’s *d* Index was calculated to evaluate the effect of size on the ANOVA and regression analyses (Table 3), and Rosenthal’s *r* was computed on the Kruskall-Wallis nonparametric test, to evaluate the degree of separation between the paired comparison means by sex or development stage: a higher value means that means are more different, i.e., more separated. In order to better understand the morphological and biochemical differences by sex or development stage, as well as differences related to the geographic distribution of the populations sampled, Principal Components Analyses (PCA) were performed with the Statistica program.

### Ethical standard

All applicable international, national, and institutional guidelines for the care and use of animals were followed.

## Results

**Figure 2:**
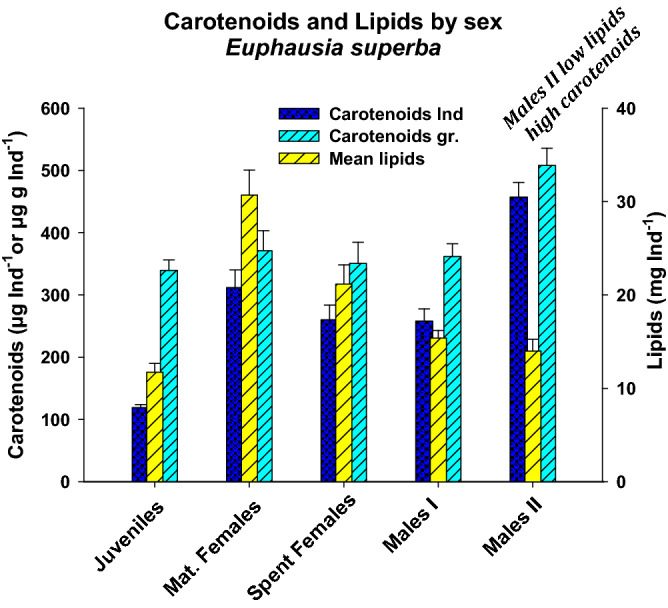
Vertical Bars chart with simple standard errors for mean lipid and carotenoid content per individual (µg Ind^−1^) and per gram (µg g^−1^, single individual) for *Euphausia superba* by sex*,* for the entire sample analysed, with standard errors.

Carotenoid levels were highest in Males II, lowest in Juveniles, and intermediate in Males I, Mature Females, and Spent Females, in either per-individual or per-g (Table 2) basis. Males II show low lipid and high carotenoid levels both per individual and per gram (Fig. 2). Carotenoid content in *E. superba* by sex showed that the morphologically different Males II group had the highest carotenoid content, both per individual and per gram (wet weight). A Kruskall-Wallis rank test showed significant differences between all groups (H^4^_114_ = 57.756, *P* < 0.001) for carotenoids per individual, and the test for the Effect of Size yielded *ƞ*^2^ = 0.4715, i.e., 47.15% of the variance is explained by the data. The Mann–Whitney test was used to test for differences between each paired combination of sex or stage groups; the results for carotenoids and lipids are shown in Table 3.

**Table 2:** Percent lipids in wet weight and per animal, carotenoids in µg per individual and µg per gram of wet weight (α = 95%); n shown in brackets in bold.

Sex or stage	% Lipids (wet weight)	Lipids mg ind^−1^	Carotenoids µg ind^−1^	Carotenoids µg g^−1^Ind ^−1^
***Euphausia superba***				
Juveniles **(44)**	2.93 ± 0.15	11.73 ± 0.93	119.19 ± 4.73	339.22 ± 16.94
Mature Females **(19)**	3.60 ± 0.23	30.71 ± 2.67	311.61 ± 28.73	370.98 ± 32.38
Spent Females **(14)**	2.91 ± 0.30	21.16 ± 2.06	260 ± 23.82	350.77 ± 33.94
Males I **(29)**	2.21 ± 0.12	15.38 ± 0.82	258.22 ± 19.47	361.84 ± 20.23
Males II **(13)**	1.54 ± 0.12	14.0 ± 1.26	457.23 ± 23.46	507.90 ± 27.73

For carotenoids by animal, a non-significant difference was found between Mature Females and Males I, between Mature Females and Spent Females, and between Spent Females and Males I; for the remaining group pairs, differences were significant (Table 3). The effect of size is shown in Table 3; Rosenthal’s r or Cohen’s d for all comparisons, particularly those including Males II, showed a clear separation of groups and a large gap. Only the comparison between Mature and Spent Females yielded a low r, i.e., a small separation between these two groups. For lipids, the Kruskall-Wallis test performed on non-normally distributed data showed significant differences between the 5 groups analysed (H^4^_114_ = 45.79, *P* < 0.001); ƞ^2^ = 0.3666 indicates that 36.66% of the variance is explained by the data; and the Mann–Whitney test for the comparison of each combination of paired groups showed significant differences between all groups, except for Males II vs. Juveniles and Males II vs. Males I, as shown in Table 3. The effect of size is also shown in Table 3 with the Rosenthal’s r value or the Cohen’s d value.

**Table 3:** Tests the biochemical variables studied with their paired comparisons with the Mann–Whitney for not normally distributed data and Student’s *t*-tests, for normally distributed data.

Comparison	Carotenoids ind^−1^		
	Mann–Whitney’s *U *or Student’s *t*	D.F.	Rosenthal’s *r* or Cohen’s *d*
Males II vs. juveniles	U = 0.0, yes (*P* < 0.001)	57	0.7161
Males II vs. males I	t = -6.447, yes (*P* < 0.001)	41	2.09, very large
Males II vs. spent females	t = -6.660, yes (*P* < 0.001)	26	2.27, very Large
Males II vs. mature females	U = 34.0, yes (*P* < 0.001)	32	0.6071
Mat. females vs. juveniles	U = 50.0, yes (*P* < 0.001)	63	0.6934
Mat. females vs. males I	t = 1.431, no (*P* = 0.159)	47	0.46, small
Mat. females vs. spent females	U = 101, no (*P* = 0.251)	32	0.2029
Spent females vs. juveniles	U = 63.0 Yes (*P* < 0.001)	58	0.5845
Spent females vs. males I	t = -0.127 No (*P*= 0.899)	42	0.02, small
Males I vs. juveniles	U = 62.0 Yes (*P* < 0.001)	72	0.7540

	Lipids (mg Ind ^−1^ )		
	Mann–Whitney’s *U *or Student’s *t*	D.F.	Rosenthal’s *r* or Cohen’s *d*
Males II vs. juveniles	t = −1.242, no (*P*= 0.219)	57	0.42, small
Males II vs. males I	U = 140.5, no (*P* = 0.251)	41	0.1816
Males II vs. spent females	U = 32, yes (*P* = 0.005)	26	0.5510
Males II vs. mat. females	U = 22, yes (*P* < 0.001)	31	0.6885
Mat. females vs. juveniles	U = 61, yes (*P* < 0.001)	62	0.6732
Mat. females vs. males I	U = 59.0, yes (*P* < 0.001)	47	0.6732
Mat. females vs. spent females	U = 67.0, yes (*P* = 0.017)	32	0.4185
Spent females vs. Juveniles	U = 103, yes (*P* < 0.001)	58	0.4918
Spent females vs. males I	U = 99.0, yes (*P* = 0.001)	42	0.3994
Males I vs. Juveniles	U = 376.5, yes (*P* = 0.004)	72	0.3365

	Carotenoids g ^−1^		
	Student’s t	D.F.	Cohen’s d
Males II vs. juveniles	4.67, yes (P< 0.001)	57	1.59 ,very large
Males II vs. males I	3.83, yes (*P* < 0.001)	41	1.40, very large
Males II vs. spent females	3.57, yes (P< 0.001)	26	1.37, very large
Males II vs. mat. females	3.34, yes (P=0.001)	32	1.12, large
Mat. females vs. juveniles	0.99, no (P=0.323)	63	0.25, small
Mat. females vs. males I	0.81, no (P=0.419)	47	0.07, small
Mat. females vs. spent females	0.49, no (P=0.624)	32	0.15, small
Spent females vs. juveniles	0.32, no (P=0.747)	57	0.10, small
Spent females vs. males I	0.29, no (P=0.771)	42	0.09, small
Males I vs. juveniles	0.27, no (P=0.791)	72	0.20 Small

Also shown is *r* for data non-normally^74^ or *d* (Cohen’s test^75^) for the normally distributed data, for the effect of size (non-overlap) for paired sex or stage groups.

For the ANOVA on carotenoids expressed as µg per g^−1^ by sex and stage (Table 1), a significant difference was observed between groups (F^4^_114_ = 5.652, *P* < 0.001), A comparison between all the possible combinations of paired sex or Stage of development groups was performed with the t of Student, all the comparisons with Males II showing significant differences, but not among the other paired comparisons. The d value for the effect of size on each paired group of data is also shown in Table 3; all the comparisons with Males II show a large or very large non-overlap according to the d value. Based on Eta (ƞ^2^_p_ = 0.8408%, D.F. = 4 and 114), 84.08% of the variance is explained by the data.

The difference between Males I and Males II, in percentage of lipid content (arc-sin transformed data) was significant. In other words, Males II had a significantly lower lipid concentration than Males I (t^1^_41_ = 3.66; *P* = 0.004) – a counterintuitive finding, since carotenoids are mostly lipid-soluble. However, on a per-individual basis, a non-significant difference was observed for lipids between the two groups (Kruskall-Wallis: H^1^_41_ = 1.126, *P* = 0.289), likely because Males I are smaller.

**Figure 3:**
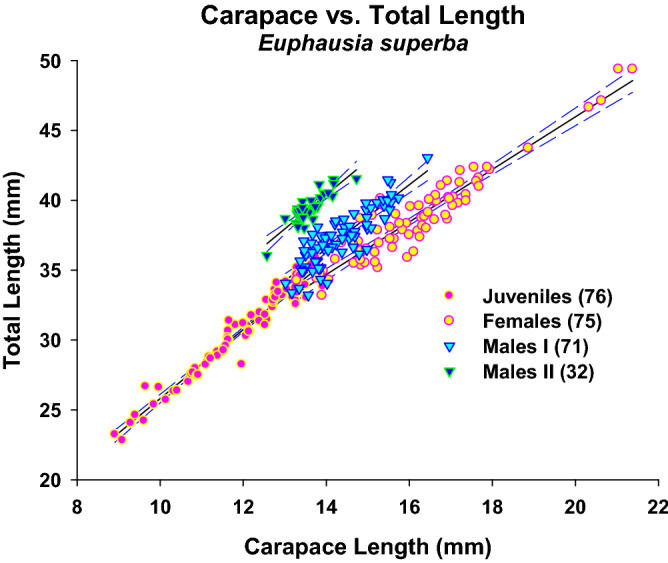
Linear regression of total length vs carapace length for all sex groups with a larger sample, with the Confidence Intervals. Data were normally distributed (Shapiro–Wilk test); but their variances were not always constant. Juveniles: TL = 1.093 + 2.471 CL, N = 76, r^2^ = 0.949, Normality: *P* = 0.276, Homoscedasticity, Failed: *P* = 0.021. Females: TL = 8.354 + 1.881CL, N = 75, r^2^ = 0.889, Normality: *P* = 0.785, Homoscedasticity, Passed: *P* = 0.115. Males I: TL = 4.417 + 2.293 CL, N = 72, r^2^   = 0.744, Normality: *P* = 0.268, Homoscedasticity, Passed: *P* = 0.328. Males II: TL = 6.162 + 2.445 CL, N = 32, r^2^ = 0.757, Normality: *P* = 0.836, Homoscedasticity, Passed: *P* = 0.245.

**Figure 4:**
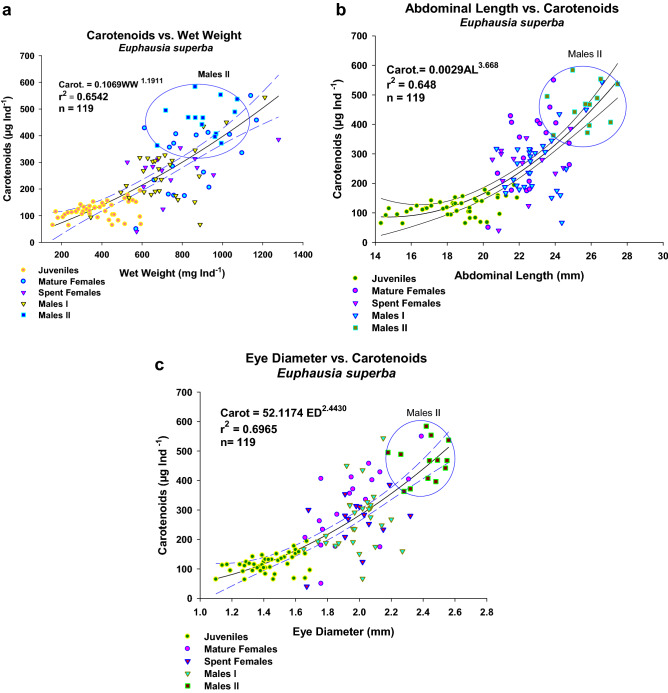
(**a**) Regression of carotenoid content per individual vs. wet weight. A power model was applied and a non-normal distribution was found (*P* = 0.0424; 95% confidence intervals included). Data for Males II encircled in blue. (**b**) Regression of carotenoid content per individual vs. abdominal length. A power model was applied and a non-normal distribution was found (*P* = 0.0470; 95% confidence intervals shown). Data for Males II encircled in blue. (**c)** Regression of carotenoid content per individual vs. eye diameter. Again, a power model was applied and a non-normal distribution was found (*P* = 0.0083; 95% confidence intervals shown). Data for Males II encircled in blue.

**Figure 5:**
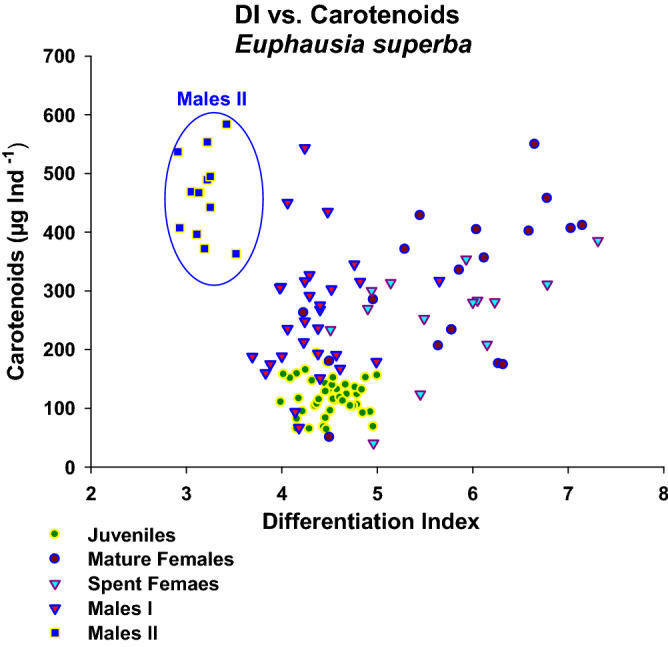
Non-significant regression between DI and carotenoids shown to highlight the distinct morphological features of Males II (Encircled in blue), clearly detached from all other sex or stage groups based on its unique body proportions.

The regression between carapace length and total length by sex or stage, analysed on a larger sample (Fig. 3), showed that Males I apparently evolved towards a shorter carapace and constitute the Males II group, given the lower significance of r^2^ in both groups. Carotenoids increased in parallel with wet weight, eye diameter, and abdominal length, as shown by the significant regressions (Fig. 4a–c). However, the data were not normally distributed, showing in all cases a clear separation of the Males II group with some mixing with the bigger Mature Females or Males I. Although the regressions between DI and carotenoids per animal for each sex or stage group (Fig. 5) were non-significant, they are shown to emphasize the morphological differences; all other regressions between the different variables by sex or development stage were non-significant due to the large individual variability. The non-significant regressions between DI and carotenoids showed that the lowest DI corresponds to the highest carotenoid concentrations in Males II, whereas higher DI values are related to lower carotenoid content. (In support of these findings, other non-significant regressions are shown in Supplementary Information 1–5).

**Figure 6:**
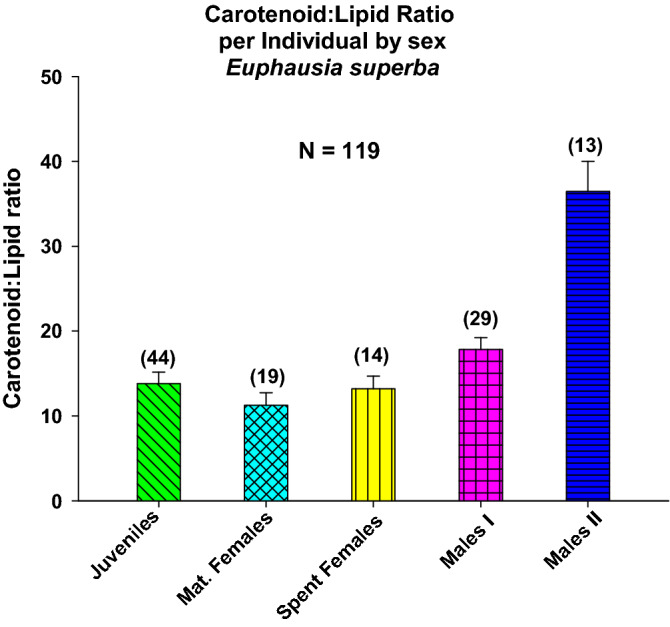
Vertical Bars chart with simple errors for the mean carotenoid:lipid ratio per individual by sex, with their standard error (α = 0.05). Number of individuals in each group (n) shown in brackets.

We used the carotenoid (µg):lipid (mg) ratio as an indicator of the physiological status of individuals (whether good or bad; this is not yet clear). It was highest for Males II and lowest for Mature Females (Fig. 6).

**Figure 7:**
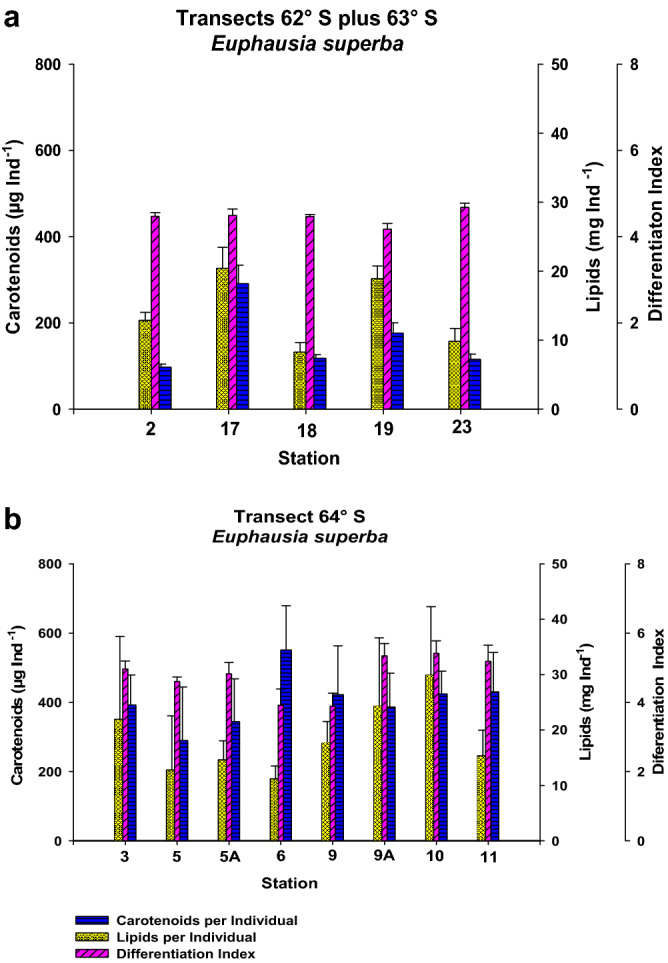
Carotenoid content by stations for *Euphausia superba*: (**a**) Transects 62°S plus 63° S. (**b**) Transect 64° S.

The analysis per transect showed that lower mean DI values are associated with a higher carotenoid content (Fig. 7a, b). The maturity of individuals in transect 64°S was markedly different relative to the other transects, being composed of adults; therefore, we decided to pool together transects 62°S and 63°S for comparison vs. transect 64°S. The pooled 62°S + 63°S transect showed lower mean carotenoid levels and intermediate and highly variable lipid values; however, the mean Differentiation Index was higher than for transect 64°S. The mean carotenoid content per gram was higher for transect 64°S (402.69 ± 14.508) than for transect 62°S + 63°S (330.22 ± 15.65), and differences were statistically significant for carotenoid content per gram (F^1^_117_ = 11.124, *P* = 0.001), mean length (Kruskall-Wallis, H^1^_117_ = 29.712, *P* < 0.001), and mean body weight (F^1^_117_ = 28.195, *P* < 0.001). Only two Male II individuals were found in transect 62°S + 63°S; however, the number of individuals for transect 62°S + 63°S was smaller (49) than for transect 64°S (70). In general, individuals were bigger and mature in transect 64°S. Also, a significant difference between transects was found for the carotenoid:lipid ratio (Kruskall-Wallis, H^1^_117_ = 6.358, *P* = 0.012), being higher for transect 64°S versus transect 62°S + 63°S (18.67 ± 1.38 vs. 14.12 ± 1.31).

**Figure 8:**
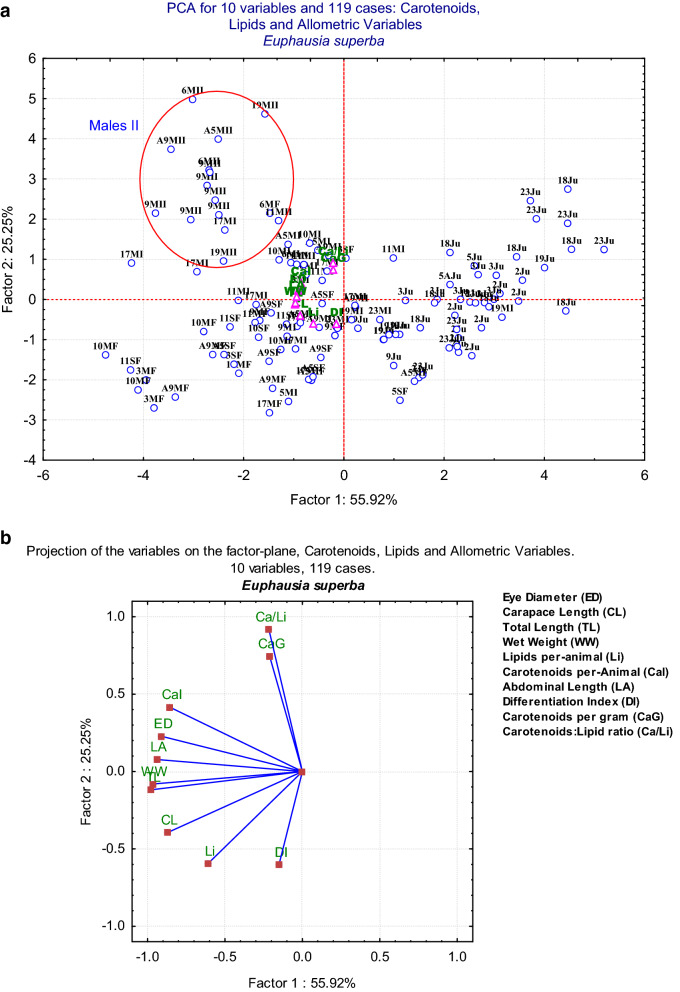
(**a**) Projection of the cases on the PCA analysis of the data analysed by station, and by sex or stage of development. A separation between sexes is evident, and again, Males II (encircled in red) forms a group clearly separated from the rest of the data. Variables are represented by pink triangles with abbreviated green letters; cases, by blue circles with their station number, and sex (Ju: Juveniles, MF: Mature Females, SF: Spent Females, MI: Males I, MII: Males II). The first two components explain 81.17% of the variance. (**b**) Biplot of the variables with their distance and direction. N = 119 with 10 active variables (in Green). All variables are positioned on the negative side of factor 1 and on both sides of Factor 2, explaining 81.17% of the variance.

When the data were analysed with a Principal Components Analysis (Fig. 8a), we found that the groups were, again, separated by sex rather than by stations or transects, probably because the differences between sexes are more important than differences between station and those associated with specific physical or trophic conditions. Males II is again separated from all other groups. However, only two of all Males II individuals were found at Station 19; all others were found at transect 64°S. The data accounted for 81.17% of the variance. The first factor was associated with the variables Wet Weight (WW), Total Length (TL), Abdominal Length (LA), and Eye Diameter (ED), and partially with Carotenoids per Individual (CAI) on the positive side, and with Carapace Length (CL) on the negative side (mostly size variables). Factor 2 is associated with Carotenoids per gram (CaG) and Carotenoid:Lipid ratio (Ca/Li) on the positive side of the axis, and with the Differentiation Index (DI) on the negative side, with lipids shared in the two axes. All Males II were grouped in a position opposite to that of the DI variable and near CaI (carotenoids per animal) and Ca/Li. The direction and distance of the variables is shown in Fig. 8b; as mentioned above, all size variables were located on the negative side of Factor 1. DI is positioned near lipids (Li), as expected, and opposite to Ca/Li and CaG; the separation of the Males II group is determined by these two variables (CaG and Ca/Li). The contributions of the ten variables are shown in Table 4.

**Table 4:** Contribution of variables to the first two factors of the PCA Analysis by sex, stage of development, and station (in italic bold values > 0.700).

Variable	Factor 1	Factor 2
Eye diameter (ED)	***− 0.912443***	0.228647
Carapace length (CL)	***− 0.873509***	− 0.395844
Total length (TL)	***− 0.976020***	− 0.118341
Wet weight (WW)	***− 0.968097***	− 0.081015
Lipids per animal (Li)	− 0.609613	− 0.595346
Carotenoids per-Animal (CaI)	***− 0.857184***	0.415815
Abdominal length (LA)	***− 0.941244***	0.077728
Differentiation index (DI)	− 0.151757	− 0.603600
Carotenoids per gram (CaG)	− 0.211853	***0.745316***
Carotenoid:lipid ratio (Ca/Li)	− 0.215939	***0.917497***
Explained variance	5.592271	2.524550
Proportion of total	0.559227	0.252455
Total first two factors	81.17%	

## Discussion

The results reported here showed that individuals with a potentially greater motile capacity, like Males II, showed a higher carotenoid content either per individual or per gram of wet weight (Table 1). This suggests that Males II probably require protecting pigments like carotenoids, or that this development stage has a faster lipid metabolism. Virtue et al. (1996)^52^ found that mature *E. superba *males had lower lipid levels, especially regarding triacylglycerol, a short-term storage lipid, during the reproductive season. Also, mature males did not survive after capture (100% death rate), contrasting with other sex groups that showed a better survival performance, such as females. From this study, these authors inferred that mortality rates in krill differ between males and females due to energetic constraints on reproduction. In our study, total lipids in Males II was equivalent to levels mentioned previously for mature males, but the role of high carotenoid levels is uncertain. Males II have bigger eyes (Fig. 4c); thus, we can assume that they accumulate more carotenoids^32,33,35^. However, the role of carotenoids in the physiology of Males II is still unclear, i.e., whether high carotenoid levels are a sign of stress or of good health. Other studies have assumed that a higher carotenoid content reflects better food quality^37,44^; however, the very low lipid levels found in Males II suggests that they are not in good physiological condition. Interestingly, carotenoid data for the total sample differs markedly when analysed on a per-individual or a per-gram basis (Table 1). This finding shows the importance of individual development stages and/or a great individual variability, probably also derived from the local trophic conditions^37,44^. An annual cycle of carotenoid composition in *Euphausia pacifica* collected from the Saanich Inlet, British Columbia, showed that the three major carotenoid pigments, making up to 85–90% of the total, are astaxanthin and its mono- and di-esters. The relative composition of the three pigments remained constant throughout the study. Temporal variations in individual and weight-specific levels appeared to be related to season and sexual maturity. These results suggested that *E. pacifica* can only store astaxanthin and its two esters in a specific ratio^37^.

The large size of *E. superba* facilitates the detection of measurable differences in the timing of spawning between males and females. We assumed that males die after fecundation and females die after spawning; this would result in a smaller proportion of males in winter and an approximately 1:1 ratio in summer^53^. This resembles the hypothesis put forward by Virtue et al.^52^, but considers females as well. A reduced swimming velocity was observed in recently spawned females^58^, which likely contributes to the segregation by sex within swarms after spawning and affects the sex ratio within a swarm.

The vertical migration on *Meganyctiphanes norvegica* was found to differ between males and females^54^; as females need more food during the reproductive season, they migrate closer to the surface to obtain it and attain reproductive success. This mechanism may also occur in the case of the Antarctic krill, *E.superba*; therefore, another possible explanation should be considered to explain the differences in sex ratio between the stations sampled in our study. The sex ratio may change in our samples simply because males were swimming deeper, and thus were not always sampled in our cruise. Although these measurements were highly variable, differences in longevity between males and females may also be an important factor^17^. Males face a high level of stress, with the probable synergistic effect of various conditions, including low lipid but high carotenoid levels and intense activity when searching for females to fertilize them, also considering that the low lipid level will not provide males with the buoyancy needed to maintain their position in the water column^29^. As a result, males would be using more energy to keep their pace at the same speed and depth as the more buoyant (because of their high lipid level) females. Under this scenario, an aspect worth investigating is whether the higher carotenoid content in Males II is an indicator of good adaptation. As mentioned by Kils^13^, krill live in a sort of physiological extreme, at the edge of their energetic capacity, which could easily lead to their death. *E. superba* can swim at speeds of more than two body lengths per second^55^ and can perform horizontal migrations, even against the prevailing current flows^56–58^; thus, its physiology is more accelerated than that of other crustaceans. The respiration rate of *E. superba* has been found to be directly proportional to individual weight, thus requiring an exponent b in the equation: Respiration rate = a weight b, where b is closer to 1 vs. other crustaceans, instead of being closer to 0.7^55^.

Krill accumulate carotenoids in the eyes^35,36,44^, a likely explanation for the higher carotenoid content in Males II, which have bigger eyes. Taken together, the higher carotenoid content, bigger abdomen, and bigger eyes seemingly provide advantages to mature Males II in search of females to fertilize. However, it is unclear whether these are really advantages or just an anticipation of their death. These findings lead to several questions: why does higher carotenoid content occur in animals with lower lipid content? Is this a consequence of a higher metabolic rate in Males II?

Another possible explanation could be the presence of carotenoproteins in crustaceans as proposed by Ceccaldi^60^ and Ceccaldi et al.^61^, which could explain the co-occurrence of low lipids and higher carotenoids. Czeczuga^62^ reported the presence of carotenoproteins in *E. superba*, but their importance and role has not been studied yet. Also, a circadian rhythm of carotenoids has been observed in the eyes and hepatopancreas of *Penaeus japonicus*^63,64^, but has never been studied in *E. superba*. Lipofuscins reflect the physiological age of an animal; they were studied by Karnaukhov^65,66^ in molluscs, and by Etteshank and others^10,67,68^ in Antarctic krill. Carotenoids are included, at least partially, in lipofuscin macromolecules, and are supposed to constitute an oxygen reserve for animals living under low-oxygen conditions^66^, but its function is still unclear in both crustaceans and molluscs. Further studies on the role of carotenoids and carotenoproteins in *E. superba* are necessary to better understand their function.

In an attempt to determine the longevity of *E. superba* using multivariate analyses, including the Differentiation Index and lipid and carotenoid content, further work by Färber Lorda^9^ sorted out morphologically different groups, with Males II clearly separated in a different cohort. Astaxanthin is the main carotenoid in krill, being a potent antioxidant^36–38^ that could provide protection when a higher metabolic rate is necessary in stages like Males II. However, krill do not synthetize carotenoids, but get them from pigment-rich food. Males II are probably feeding well, but their faster metabolic rate and fecundation needs preclude the accumulation of lipids. A work by Auerswald et al.^41^ described changes in pigment content between summer and winter, concluding that carotenoids serve as protection from solar radiation and are adapted to the changing seasonal and diel light conditions. In our study, during summer and away from the ice edge, animals need additional protection provided by pigments like carotenoids. However, in this paper we found that high carotenoid levels are apparently related not only to light intensity, but also to the activity of animals. We still do not know if the carotenoid levels observed in this study are typical during summer, when animals are not under the pack ice, nor the role of these higher carotenoid levels in Males II.

Most regression analyses were non-significant; however, the different regressions, whether non-significant or significant, demonstrate the uniqueness of the Males II group; they do show that this group differentiates from all others in either morphological or biochemical variables. Our sampling took place during the summer and over a relatively short period of time; thus, it reflects summer conditions. In Males II, carotenoid content was higher in both per-individual and per-gram basis. The Cohen factor for the effect of size on the data showed differences in per-gram carotenoid content between Males II and all other sex groups or stages; Males II was the statistically significant different group in all cases, with the lowest overlap on the data, according to the Cohen’s *d* test. The approach in this study is spatial, not seasonal. The significant differences found between transects in terms of total length, wet weight, total carotenoids per individual, and the carotenoid:lipid ratio evidence a major role of carotenoids in mature individuals, with higher mean values for transect 64°S likely related to the reproductive condition and the greater motility associated with it. However, when the Principal Components Analysis was performed on the data by station, groups were separated only for the different sex or stage groups, but not by transect or station. Males II were present only at the southernmost transect, except for two individuals in Station 19. This station was located at the rim of a gyre, which may have caused some mixing with the other transects^12^. Males II formed a clearly distinct group (Fig. 7b). The analysis by transect is hard to interpret, since each station shows individuals with markedly different characteristics, mainly as regards differences between sex and development stages. For example, lipids and their corresponding DI have opposite values, i.e., high values in Females and low in Males II, with both present in the same stations regardless of the local conditions. Nonetheless, the separation of the Males II group was evident. The PCA analysis shows the uniqueness of the Males II group, with 10 variables included, and determine the unique characteristics of this group. The variables that best determine the group are the Carotenoid:Lipid ratio and the carotenoid content per gram, and the fact that Males II are placed on the opposite side of the variable DI, which determines the body proportions in each group of sex or development stage.

Various aspects of our study point to the greater motility of Males II, probably in search of females for fecundation, including the bigger abdomen and larger eye diameter, which translate into higher accumulation of carotenoids^35,43^. This group showed a unique morphometry in all the regressions analysed (see also SI 1–5), in addition to higher carotenoid and lower lipid contents. The carotenoid:lipid ratio is highest for Males II and lowest for Mature Females, another indication of a unique physiology in Males II. These findings, together with the lower DI in Males II (Table 1), support the hypothesis of a central role of carotenoids in the physiology of Male II individuals, since a lower DI means more active animals if we follow the hypothesis of a greater activity level in Males II.

Geographically, samples collected in the southern transect had a significantly higher carotenoid content per gram, and were composed of more mature individuals, including a higher number of Males II, characterised by significantly greater total length and wet weight, and a greater mean carotenoid:lipid ratio, all of which are likely related to greater motility. A high DI is associated with lower carotenoid content per individual, especially in Males I. There is no proof of a direct relationship between lower DI and higher carotenoid content, but an interesting relationship is shown. Using data normalised by weight, a higher respiration rate was recently found in mature male individuals that show DI values lower than 3.5 (Tarling et al., in preparation), which supports our findings. A comparison of the three most important species of krill was conducted, showing that *E. superba* has the greatest carapace and pleopod length relative to the other species studied, as well as a reduction of carapace length relative to total length in males (Tarling et al., submitted). This study suggests a potential relationship of carotenoids with the activity of individuals. The implication of these findings are that a lower DI is equivalent to a greater energetic expenditure, which probably explains our results.

### Hypotheses

- During the reproductive season, Males II are under stress associated with reproduction. They are more active when searching for females, and use an excess energy that is not compensated for by food intake. As a result, Males II show a low lipid content and higher carotenoid levels (which could be present as carotenoproteins;^60,61^), the latter functioning as protective pigments derived from the accelerated metabolism in this development stage. Males II die after fecundation, as proposed by other authors^38,53^.
- The biochemical composition of Males II reflects an accelerated metabolism during the reproductive season, with lower lipid but higher carotenoid levels, the latter being the protective pigment for an accelerated metabolism; animals subsequently recover in the rich summer environment when primary production is high, re-maturing repeatedly^69–71^.
- A combination of the two hypotheses above: krill will die if they do not encounter favourable trophic conditions, or they will re-start the maturation process when they find favourable trophic conditions, during their continuous displacements by either active swimming or advection (which may also play a role in these scenarios, with less energy expenditure when advected). Krill will make good use of the energy-saving formation of swarms^72,73^, considering the characteristics of this highly adaptive species that shows an outstanding plasticity in its adaptions^18,59^.

## Conclusions

In *E. superba*, the Males II group showed a unique morphometry and always appeared as a separate group in all the signficant and  non-significant regressions plotted.

The morphologically unique Males II also showed higher carotenoid and lower lipid contents, both per individual and per gram of wet weight. Geographically, population composition plays an important role in total carotenoid and total lipid contents, which depend on their particular morphology and reflect different swimming capacities.

The large size of *E. superba* allows obtaining measurable differences between individuals and sexes on both morphometric and biochemical composition. The high carotenoid:lipid ratio reflects the unique physiology of Males II and the likely protective role of carotenoids in euphausiids.

The Principal Components Analysis on 10 variables shows mostly differences between sex stages in morphometrics and the biochemical composition of individuals, particularly for Males II.

In view of previous results, we consider that the most realistic hypothesis to explain the higher carotenoid and lower lipid contents observed in our study is seemingly that of stressed animals that will die after fecundation.

The carotenoid:lipid ratio shows higher values in Males II, suggesting a distinctive physiological profile during the reproductive season.

However, too many uncertainties remain, limiting the strength of the conclusions about the physiological role of the higher carotenoid content in Males II.

## Supplementary information

Supplementary Information

## Data Availability

All data are available from the authors upon request.
