# Supplementary material for: Relationship of morphometrics, total carotenoids, and total lipids with activity and sexual and spatial features in Euphausia superba
Source: Sci Rep. 2020 Aug 6;10:13177. doi: 10.1038/s41598-020-69780-8 (PMC7411069; doi:10.1038/s41598-020-69780-8)
Supplement: Supplementary file 1 — Supplementary Information [file 41598_2020_69780_MOESM1_ESM.pdf]

Supplementary Information for:

Relationship of Morphometrics, Total Carotenoids, and Total Lipids with Activity and Sexual and Spatial Features in *Euphausia superba.*

Färber Lorda, J., & Ceccaldi, H. J.

Supplementary Material

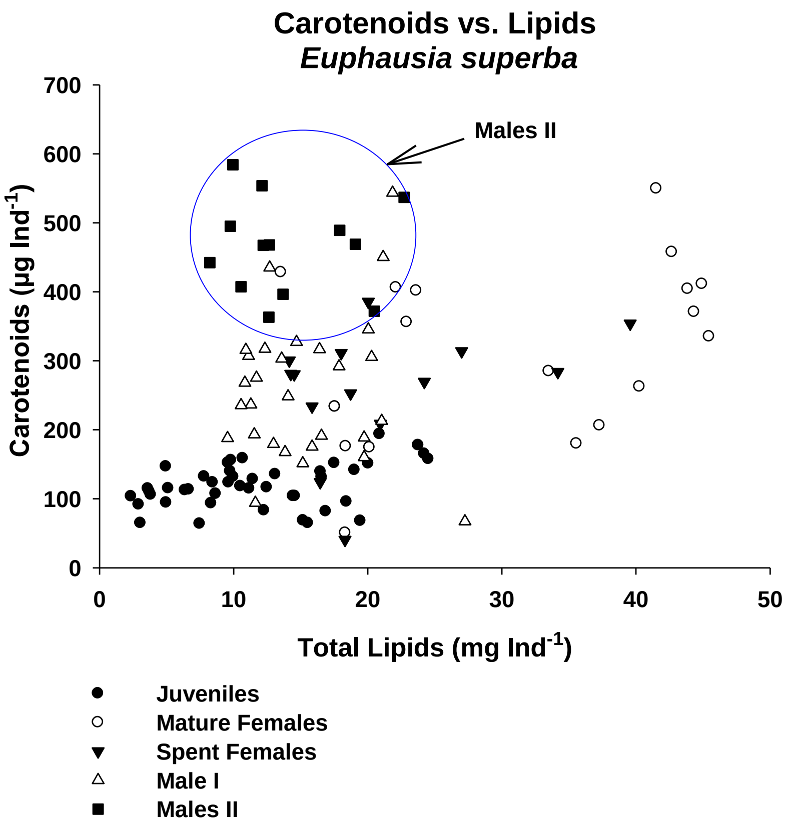

Supplementary Fig. 1 The non- significant regression between total lipids and carotenoids per sex and stage of development. Higher carotenoids and lower lipids separate the Males II groups from the rest. Data were not normally distributed.

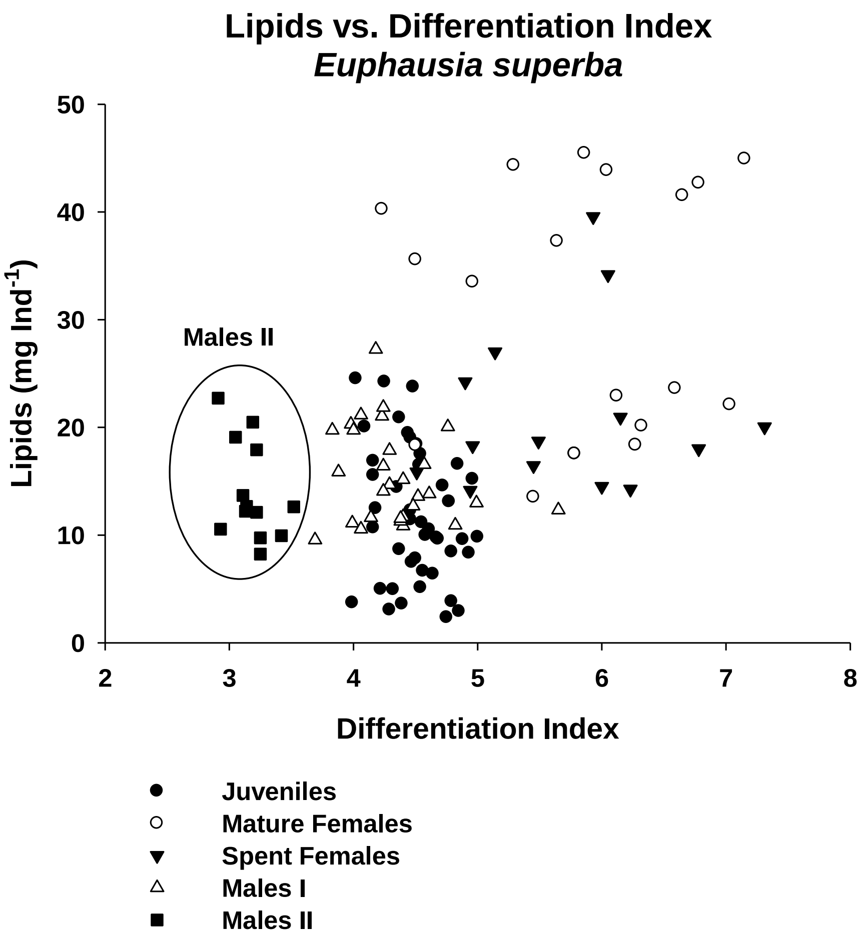

Supplementary Fig. 2.- The not significant regression between the Differentiation Index and lipids, by sex or stage of development. Data were non-normally distributed. Males II are separated from the other groups of sex or development stage, due to their low lipid contents.

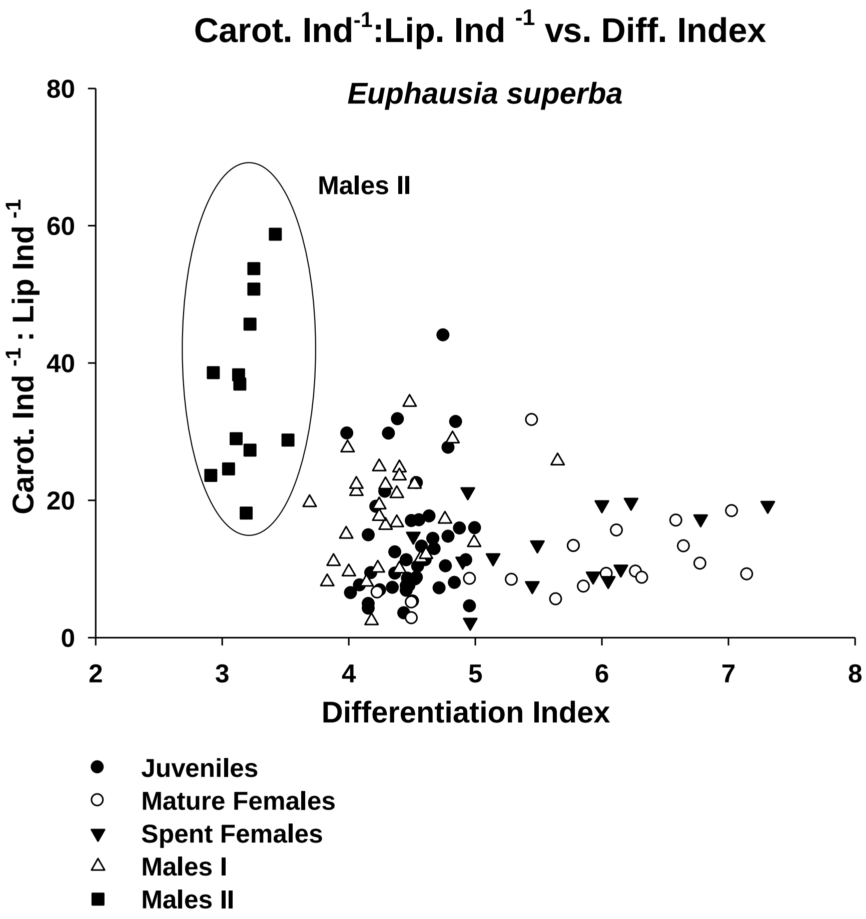

Supplementary Fig. 3.- The not significant regressions between the Carotenoids : lipids ratio per animal and the Differentiation Index, by sex or stage of development. Males II group is clearly detached from the other groups. Data were non-normally distributed.

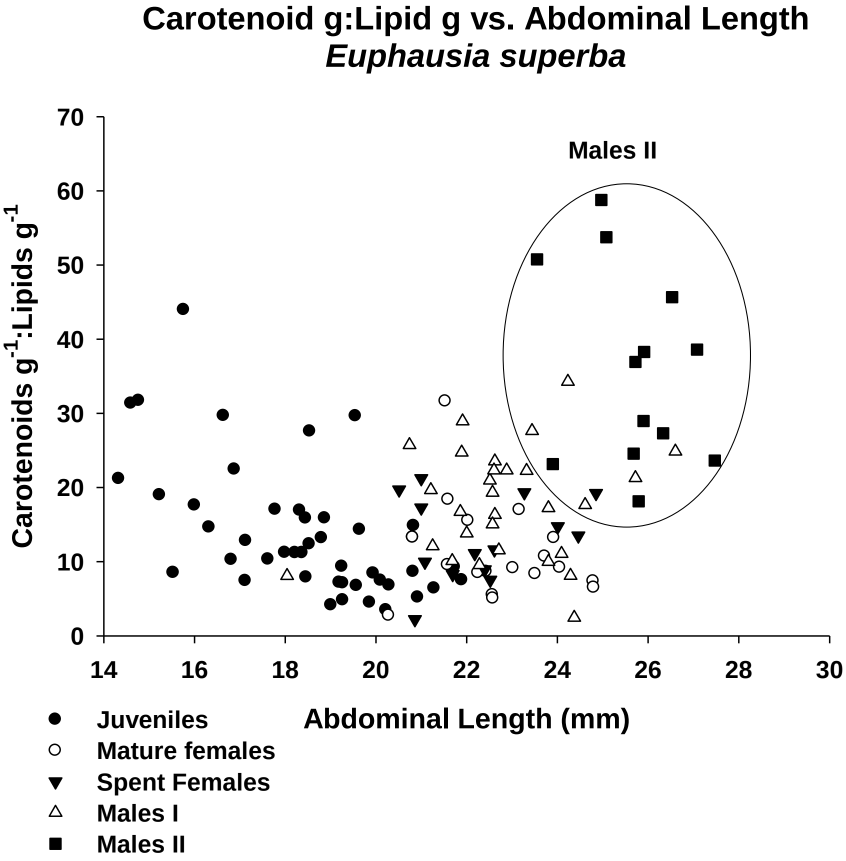

Supplementary Fig. 4.- The not significant regressions between Carotenoid g:lipids g vs. Abdominal Length. Again Males II is detached from the other groups.

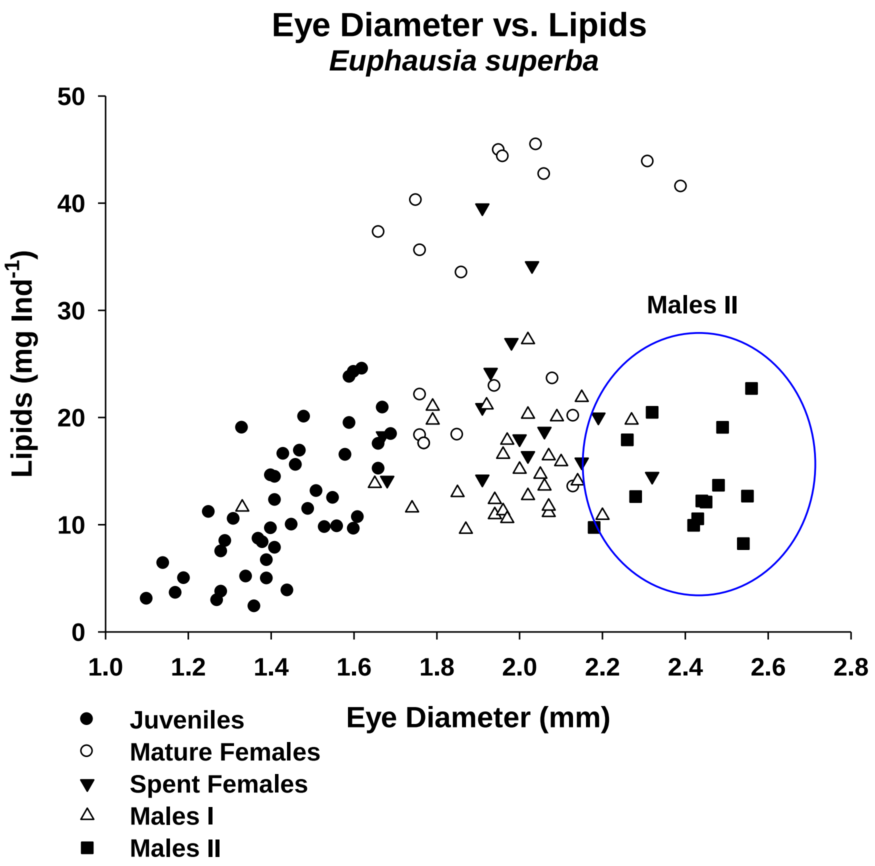

Supplementary Fig. 5.- The not significant regression between Eye Diameter and Lipids. Data were not normally distributed. Due to their greater eyes, Males II are detached from the rest of the groups.
